# Burden of congenital anomalies in Faisalabad, Pakistan: Consanguinity, ethnic disparities and public health implications

**DOI:** 10.12669/pjms.41.12.12694

**Published:** 2025-12

**Authors:** Sidra Sajid, Zulaikha Batool, Areesha Rashid, Saima Naz, Sajid Malik

**Affiliations:** 1 Sidra Sajid, Human Genetics Program, Department of Zoology, Quaid-i-Azam University, Islamabad, Pakistan; 2 Zulaikha Batool, Human Genetics Program, Department of Zoology, Quaid-i-Azam University, Islamabad, Pakistan; 3 Areesha Rashid, Human Genetics Program, Department of Zoology, Quaid-i-Azam University, Islamabad, Pakistan; 4 Saima Naz, Department of Anatomy, Rawalpindi Medical University, Rawalpindi, Pakistan; 5 Sajid Malik, Human Genetics Program, Department of Zoology, Quaid-i-Azam University, Islamabad, Pakistan

**Keywords:** Birth defects, Clinico-epidemiological study, Hereditary anomalies, Neurological disorders, Sporadic

## Abstract

**Objective:**

Congenital anomalies (CA) have emerged as a leading cause of infant morbidity and mortality worldwide, particularly in low-resource countries such as Pakistan. This study investigated the distribution, types, and biodemographic attributes of CA in the Faisalabad Division of Punjab, Pakistan.

**Methodology:**

A clinico-epidemiological study was conducted from September 2023 to April 2024. Individuals and families with CA were recruited through multiple approaches, including visits to Allied hospital, special education centers, Sundus Foundation and random door-to-door surveys. The anomalies were classified with the help of clinicians using the *OMIM* and *ICD-10* databases. Descriptive statistics were employed for data analysis.

**Results:**

A total of 867 independent individuals/families with CA were included. The CA were classified into 12 major and 107 minor categories. Among these major categories, neurological disorders were the most prevalent (40%), followed by sensorineural/ear defects (13%), neuromuscular disorders (9%), limb defects (7%), blood disorders (6%), orofacial defects (4%), musculoskeletal disorders (4%), congenital heart defects (4%), eye/visual impairments (4%), urogenital defects (3%), metabolic defects (3%) and others (2%). The parental consanguinity was observed in 62% cases. There was remarkably high representation of sporadic cases (66%) compared to familial (34%), respectively. Marked differentials were evident in the distribution of CA across the demographic, socio-economic and ethnic attributes of individuals.

**Conclusions:**

Majority of the anomalies observed in this cohort are of severe nature suggesting the need for targeted public health interventions. There is a dire need for awareness programs regarding maternal exposures and parental consanguinity and prenatal screening and genetic counselling services.

## INTRODUCTION

In recent decades, infectious diseases were considered the leading cause of infant mortality. However, the introduction of new antibiotics, advancements in immunology and improvements in preventive care have shifted this trend, making congenital anomalies (CA) a more prevalent cause of morbidity and infant death.^1^ CA are defined as structural or functional anomalies of fetal origin that are present at birth and may affect an individual’s health, development and/or survival.^2^ CA pose a significant public health burden because they are associated with physical, intellectual, developmental and other health issues. Every year, approximately 303,000 newborns die from CA within the first four weeks of life.^3^,^4^ Globally, CA account for around 9% of all deaths in children under five, and those who survive often face physical disabilities, stigma, social isolation and lifelong impairments.^5^

With a child mortality rate of 65.2 per 1,000 births, Pakistan has one of the highest under-five mortality rates globally, significantly exceeding the global average of 37 deaths per 1,000 live births as of 2020.^6^ CA account for 2-9% of total deaths in Pakistan.^4^,^7^ This high burden is attributed to several factors, including a high rate of consanguineous marriages, large family sizes, low socioeconomic status and various maternal factors.^7^ This situation is further exacerbated by systemic challenges including a scarcity of human resources, imbalanced allocation of healthcare workers and inadequate maintenance of rural health facilities.^7^ Despite the significant impact of CA, large population-based studies in Pakistan are lacking, although several hospital-based studies have been reported.^8^

The Faisalabad division is a major metropolitan in Pakistan; however, no such study is available on the distribution and burden of CA in this population. Towards this end, the current study aims to provide insights into the epidemiology of CA in the Faisalabad division.

## METHODOLOGY

A cross-sectional and clinico-epidemiological study on CA was conducted from September 2023 to April 2024, in the Faisalabad division of Punjab, Pakistan. Data collection was carried out using a combination of methods, including door-to-door visits, hospital-based surveys (Allied Hospital; Sundus Foundation), specialized clinic surveys and snowball sampling. Pre-diagnosed cases of CA registered at various special education centers were also included. Information on demographic characteristics and family history were collected using a structured proforma in face-to-face interview. Only the index subject from each family was included in the primary data analysis. Sample size for rare disorders was calculated through sample size formula for proportion estimation.

### Classification of anomalies and statistical analysis:

Physical examinations and primary diagnoses of CA were conducted by pediatricians and clinical specialists. For further clarification, previous medical records of the patients were also reviewed. The ICD-10 and OMIM databases were used for classification and confirmation of congenital anomalies.

### Ethical Approval:

This study was approved by the Ethical Review Committee of Quaid-i-Azam University (DAS-15; dated: July 3, 2019) and all participants were recruited in accordance with the Declaration of Helsinki. Before collecting personal information, all participants or their parents/guardians were assured that no ethical breaches would occur. Written informed consent was obtained from parents or legal guardians of the participants, as most index subjects were minors or unable to consent due to disabilities.

### Inclusion & Exclusion Criteria:

Only the individuals with a confirmed diagnosis of CA were included, while those with anomalies of traumatic origin or infections were excluded. The CA were resolved into major categories based on the involvement of major organ system. A detailed pedigree was constructed for each case.

### Statistical Analysis:

All data were maintained in MS-Excel and analyses were carried out through GraphPad Prism (Version 5.00). Descriptive summaries were generated and index cases were classified based on gender, familial/sporadic occurrence and isolated/syndromic nature. For CA, proportions and corresponding 95% confidence intervals (CI) were calculated.

## RESULTS

### Sample characteristics:

The study included 867 index participants representing independent families. Male index cases constituted 62%, while females accounted for 38% (Table-I). Overall, 82% of participants were aged ≤19 years. Detailed demographic characteristics are shown in Table-I.

**Table-I:** Demographic attributes of index cases and odds of affected males.

Variable	Male, n (%)	Female, n (%)	Total, n (%)	Proportion	95% CI	OR#
***District***						
Toba Tek Singh	310 (58)	164 (50)	474 (55)	0.547	0.514-0.580	Ref.
Faisalabad	195 (36)	140 (43)	335 (39)	0.386	0.354-0.419	0.74
Others	34 (6)	24 (7)	58 (7)	0.067	0.050-0.084	0.75
***Origin***						
Rural	389 (72)	228 (70)	617 (71)	0.712	0.681-0.742	1.14
Urban	150 (28)	100 (30)	250 (29)	0.288	0.258-0.319	Ref.
***Age ranges (years)***						
Up to 9	246 (46)	154 (47)	400 (46)	0.461	0.428-0.495	Ref.
>9-19	191 (35)	118 (36)	309 (36)	0.356	0.325-0.388	1.01
≥19	102 (19)	56 (17)	158 (18)	0.182	0.157-0.208	1.14
***Language***						
Punjabi	420 (78)	249 (76)	669 (77)	0.772	0.744-0.800	Ref.
Urdu	96 (18)	66 (20)	162 (19)	0.187	0.161-0.213	0.86
Others	23 (4)	13 (4)	36 (4)	0.042	0.028-0.055	1.05
***Caste-system***						
Jatt	115 (21)	64 (20)	179 (21)	0.206	0.180-0.233	Ref.
Arain	93 (17)	52 (16)	145 (17)	0.167	0.142-0.192	1.00
Rajput	76 (14)	48 (15)	124 (14)	0.143	0.120-0.166	0.88
Malik	25 (5)	15(5)	40 (5)	0.046	0.032-0.060	0.93
Sheikh	24 (4)	15 (5)	39 (4)	0.045	0.031-0.059	0.89
Others	206 (38)	134 (41)	340 (39)	0.392	0.360-0.425	0.86
***Economic status*** *						
Low	362 (67)	220 (67)	582 (67)	0.671	0.640-0.703	2.54
Mid	166 (31)	91 (28)	257 (30)	0.296	0.266-0.327	2.82
High	11 (2)	17 (5)	28 (3)	0.032	0.021-0.044	Ref.
Total	539 (62)	328 (38)	867 (100)	1.000	-	

* p=0.031; Ref.= reference; #Odd ratios of affected males compared to females.

### Classification of congenital anomalies:

The CA were classified into 12 major and at least 107 minor entities (Tables II-III). Among the major categories, neurological disorders were the most prevalent, accounting for 40% of cases, followed by sensorineural/ear defects (13%), neuromuscular disorders (9%), limb defects (7%), blood disorders (6%), orofacial defects (4%), musculoskeletal disorders (4%), congenital heart defects (4%), eye/visual impairments (4%), urogenital defects (3%), metabolic defects (3%) and others (2%) (Table-II).

**Table-II:** Major categories of CA, familial/sporadic occurrence and parental consanguinity.

Anomaly	Total, n (%)	Gender, n (%) *		Familial/sporadic nature, n (%) **		Parental consanguinity, n (%) ***
		Male	Female	Familial	Sporadic	
Neurological disorders	344 (40)	210 (39)	134 (41)	109 (32)	235 (68)	194 (56)
Sensorineural/ear defects	111 (13)	76 (14)	35 (11)	52 (47)	59 (53)	78 (70)
Neuromuscular disorders	81 (9)	50 (9)	31 (9)	25 (31)	56 (69)	49 (60)
Limb defects	64 (7)	37 (7)	27 (8)	24 (38)	40 (62)	40 (62)
Blood disorders	52 (6)	33 (6)	19 (6)	26 (50)	26 (50)	43 (83)
Orofacial defects	38 (4)	24 (4)	14 (4)	11 (29)	27 (71)	25 (66)
Musculoskeletal disorders	38 (4)	22 (4)	16 (5)	12 (32)	26 (68)	24 (63)
Congenital heart defects	35 (4)	18 (3)	17 (5)	3 (9)	32 (91)	19 (54)
Eye/visual impairments	34 (4)	18 (3)	16 (5)	15 (44)	19 (56)	23 (68)
Urogenital defects	27 (3)	27 (5)	0	1 (4)	26 (96)	17 (63)
Metabolic defects	23 (3)	15 (3)	8 (2)	6 (26)	17 (74)	17 (74)
Others	20 (2)	9 (2)	11 (3)	7 (35)	13 (65)	12 (60)
Total	867 (100)	539 (62)	328 (38)	291 (34)	576 (66)	541 (62)

* p=0.0094;

** p<0.0001;

*** p=0.0402 (consanguineous vs. non-consanguineous).

**Table-III:** Major and minor categories of congenital anomalies.

Major/minor categories	Frequency	Proportion	95% CI	ICD-10	OMIM
***Neurological disorders***	344	0.397	0.364-0.429		
Intellectual disability (ID) types	232	0.268	0.238-0.297	249500	F79
Mild ID	105	0.121	0.099-0.143		F70
Moderate ID	64	0.074	0.056-0.091		F71
Severe ID	57	0.066	0.049-0.082		F72
Profound ID	6	0.007	0.001-0.012		F73
Down syndrome	43	0.050	0.035-0.064	190685	Q90.9
Developmental delay	17	0.020	0.010-0.029	618330	F88
Epilepsy (juvenile myoclonic)	16	0.018	0.009-0.027	604364	G40.3
Hydrocephalus	10	0.012	0.004-0.019	236600	Q03.9
Myelomeningocele	9	0.010	0.004-0.017	182940	Q05.9
Spina bifida	7	0.008	0.002-0.014	182940	Q05.9
Microcephaly	6	0.007	0.001-0.012	251200	Q02
Bardet-Biedl syndrome	1	0.001	-0.001-0.003	209900	Q87.89
Fragile-X syndrome	1	0.001	-0.001-0.003	300624	Q99.2
MASA syndrome	1	0.001	-0.001-0.003	303350	Q87.8
Tethered cord syndrome	1	0.001	-0.001-0.003		Q06.8
***Sensorineural/ear defects***	111	0.128	0.106-0.150		
Deaf and mute	103	0.119	0.097-0.140	304500	H90
Mute	4	0.005	0.000-0.009		R47.0
Stuttering	2	0.002	-0.001-0.006	184450	F98.5
Accessory tragus	1	0.001	-0.001-0.003		Q17.0
Microtia	1	0.001	-0.001-0.003	600674	Q17.2
***Neuromuscular disorders***	81	0.093	0.074-0.113		G70-G73
Cerebral palsy (CP) types	81	0.093	0.074-0.113	612900	G80-G83
**Spastic CP**	26	0.030	0.019-0.041		G80.0
Spastic quadriplegia	13	0.015	0.007-0.023		G80.0
Spastic hemiplegia	5	0.006	0.001-0.011		G80.2
Spastic monoplegia	5	0.006	0.001-0.011		G83.1
Spastic diplegia	3	0.003	0.000-0.007		G80.1
**Athetoid CP**	27	0.031	0.020-0.043		G80.3
Athetosis	21	0.024	0.014-0.034		R25.8
Dystonia	6	0.007	0.001-0.012		G24.9
**Ataxic CP**	19	0.022	0.012-0.032		G80.4
**Unspecified CP**	9	0.010	0.004-0.017		G80.9
***Limb defects***	64	0.074	0.056-0.091		
Talipes types	23	0.027	0.016-0.037	119800	Q66.6
Polydactyly types	22	0.025	0.015-0.036	174400	Q69.1
Postaxial polydactyly	17	0.020	0.010-0.029	174200	Q69.0
Preaxial polydactyly	5	0.006	0.001-0.011	174400	Q69.1
Brachydactyly	3	0.003	0.000-0.007	113300	Q68.1
Cleft-hand-foot	2	0.002	-0.001-0.006	183600	Q71.6
Radial hemimelia	2	0.002	-0.001-0.006	114500	Q71.4
Syndactyly (fingers)	2	0.002	-0.001-0.006	610713	Q70.9
Amputation (transverse) of foot	1	0.001	-0.001-0.003	217100	Q72.0
Brachydactyly-symphalangism syndrome	1	0.001	-0.001-0.003	113450	
Club thumb	1	0.001	-0.001-0.003	113200	R68.3
Constriction ring	1	0.001	-0.001-0.003	217100	Q79.8
Hallux valgus	1	0.001	-0.001-0.003		M20.1
Macrodactyly	1	0.001	-0.001-0.003	155500	Q74.2
Oligodactyly	1	0.001	-0.001-0.003	176240	Q73.8
Thumb hypoplasia	1	0.001	-0.001-0.003		Q71.6
Zygodactyly	1	0.001	-0.001-0.003	609815	Q70.1
Symbrachydactyly	1	0.001	-0.001-0.003	113000	
***Blood disorders***	52	0.060	0.044-0.076		
Thalassemia major	41	0.047	0.033-0.061	613985	D56
Haemophilia A	9	0.010	0.004-0.017	306700	D66
Fanconi anemia	1	0.001	-0.001-0.003	227650	D61.09
von Willebrand disease	1	0.001	-0.001-0.003	193400	D68.0
***Musculoskeletal disorders***	38	0.044	0.030-0.057		
Muscular dystrophy	12	0.014	0.006-0.022	310200	G71.0
Skeletal dysplasia	8	0.009	0.003-0.016	618870	Q79
Muscle hypotonia	6	0.007	0.001-0.012		P94.2
Achondroplasia	3	0.003	0.000-0.007	100800	Q77.4
Craniofrontonasal dysplasia	3	0.003	0.000-0.007	304110	Q75.9
Hip hypoplasia	2	0.002	-0.001-0.006	602484	Q74.2
Kyphoscoliosis	1	0.001	-0.001-0.003	610170	M41
Marfan syndrome	1	0.001	-0.001-0.003	154700	Q87.4
Midfacial hypoplasia	1	0.001	-0.001-0.003		Q87.0
Rickets	1	0.001	-0.001-0.003	100800	E34.4
***Orofacial defects***	38	0.044	0.030-0.057		
Cleft lip and palate	16	0.018	0.009-0.027	119530	Q37.0
Cleft palate	14	0.016	0.008-0.025	119540	Q35
Cleft lip	5	0.006	0.001-0.011	119530	Q36
Macrostomia	1	0.001	-0.001-0.003	613545	Q18.4
Pierre Robin sequence	1	0.001	-0.001-0.003	261800	Q87.0
Tongue-tie	1	0.001	-0.001-0.003	106280	Q38.1
***Congenital heart defects (CHD)***	35	0.040	0.027-0.053	614954	Q23.4
Ventricular septal defect	10	0.012	0.004-0.019	614429	Q21.0
Cardiomegaly	2	0.002	-0.001-0.006		I51.7
Tetralogy of Fallot	2	0.002	-0.001-0.006	187500	Q21.3
Pulmonary vein stenosis	1	0.001	-0.001-0.003		Q26.4
Unspecified CHD	20	0.023	0.013-0.033		Q24.9
***Eye/visual impairments***	34	0.039	0.026-0.052		
Congenital cataract	15	0.017	0.009-0.026	115700	Q12.0
Blindness	8	0.009	0.003-0.016	216900	H54.9
High myopia	3	0.003	0.000-0.007	160700	H52.10
Congenital ptosis	2	0.002	-0.001-0.006	300245	Q10.0
Strabismus	2	0.002	-0.001-0.006	185100	H50.9
Amblyopia	1	0.001	-0.001-0.003		H53.0
Anophthalmia	1	0.001	-0.001-0.003	251600	Q11.2
Congenital epiphora	1	0.001	-0.001-0.003	149700	Q10.5
Microphthalmia	1	0.001	-0.001-0.003	251600	Q11.2
***Urogenital defects***	27	0.031	0.020-0.043		
Hypospadias	21	0.024	0.014-0.034	300633	Q54.1
Epispadias	4	0.005	0.000-0.009	600057	Q64.0
Bartter syndrome	1	0.001	-0.001-0.003	241200	E26.8
Bladder exstrophy	1	0.001	-0.001-0.003	600057	Q64.1
***Metabolic defects***	23	0.027	0.016-0.037		
Cystic fibrosis	6	0.007	0.001-0.012	219700	E84.0
Galactosemia	5	0.006	0.001-0.011	230200	E74.2
Mucopolysaccharidosis	4	0.005	0.000-0.009	252800	E76.0
Congenital adrenal hyperplasia	3	0.003	0.000-0.007	201910	E25.0
Hypothyroidism	2	0.002	-0.001-0.006	218700	E03.1
Wilson disease	2	0.002	-0.001-0.006	277900	E83.0
Gaucher disease	1	0.001	-0.001-0.003	230800	E75.2
***Others***	20	0.023	0.013-0.033		
Celiac disease	9	0.010	0.004-0.017	212750	K90.0
Albinism	2	0.002	-0.001-0.006	203100	E70.3
Allergic rhinitis	1	0.001	-0.001-0.003		J30.9
Alopecia	1	0.001	-0.001-0.003	104000	L63.9
Cutis aplasia congenita	1	0.001	-0.001-0.003	600360	Q84.8
Dermoid cyst (periocular)	1	0.001	-0.001-0.003		D31.0
Ichthyosis	1	0.001	-0.001-0.003	242300	L85.0
Neonatal cholestasis	1	0.001	-0.001-0.003	211600	P59.1
Sjögren syndrome	1	0.001	-0.001-0.003	270150	M35.0
Tracheoesophageal fistula	1	0.001	-0.001-0.003	189960	Q39.2
Umbilical hernia	1	0.001	-0.001-0.003		K42

Among the neurological disorders, there was the highest representation of intellectual disability (26.8%), which could be further categorized into mild, moderate, severe and profound types (Table-III). The cases were Down syndrome accounted 5%, followed by developmental delay (2%) and juvenile myoclonic epilepsy (1.8%). Among the patients with sensorineural/ear defects, deaf-mute cases were most common (11.9%); among the neuromuscular disorders, spastic cerebral palsy and athetoid cerebral palsy were most prominent (3% and 3.1%, respectively). In limb defects, there were 17 minor entities and talipes and polydactyly were most common (2.7% and 2.5%, respectively). Thalassemia major had the highest representation in blood disorders (4.7%). There were several rare disorders represented in 1 or 2 patients (Table-III).

### Familial/sporadic nature, affected sex ratio, parental consanguinity:

Most anomalies occurred sporadically (66%) rather than in familial clusters (34%) (Table II). Sporadic cases were particularly common in congenital heart defects (91%), urogenital anomalies (86%), metabolic disorders (74%), and orofacial defects (71%). In contrast, familial occurrence was more frequent in blood disorders (50%), sensorineural/ear defects (47%), and eye/visual impairments (44%) (p < 0.001).

Pedigree analyses showed that across all families, there were 1393 affected individuals (males 62%, female 38%) and the differences in the distribution of males and females was statistically significant (p<0.0001) (data not shown). Parental consanguinity was reported in 62% of cases, highest among blood disorders (83%), followed by metabolic (74%), sensorineural/ear (70%), and eye/visual impairments (68%) (p = 0.04) (Table-II).

### Disparities in demographic and ethnic distribution of congenital anomalies:

Significant regional and demographic variations were observed in the distribution of major CA categories (Table-IV). In the Toba Tek Singh tehsil, neurological, sensorineural/ear, and neuromuscular disorders were more prevalent than in other regions. Patients with sensorineural/ear or neuromuscular disorders were predominantly of rural origin. Neurological disorders mainly affected participants aged >9–19 years, whereas blood and orofacial anomalies were more frequent in younger children (up to 9 years). Neurological disorders were most common among Punjabi-speaking participants. Statistically significant differences were also observed across caste and socioeconomic groups (Table-IV).

**Table-IV:** Differences in the distribution of major categories of congenital anomalies across demographic variables

Variable*	Neurological disorders	Sensorineural/ear defects	Neuromuscular disorders	Limb defects	Blood disorders	Musculoskeletal disorders	Orofacial defects	Others
***District***								
Toba Tek Singh	46	19	15	7	0	5	1	5
Faisalabad	34	4	3	8	13	2	9	28
Others	24	12	2	7	12	7	3	33
Total	40	13	9	7	6	4	4	16
***Origin***								
Rural	38	16	10	8	4	5	4	15
Urban	44	6	7	7	10	4	5	18
***Age ranges (years)***								
Up to 9	33	6	7	6	9	4	9	26
>9-19	51	18	10	6	4	2	-	9
≥19	35	22	13	13	2	9	1	4
***Language***								
Punjabi	41	14	11	7	4	5	4	14
Urdu	37	7	6	7	14	-	7	22
Others	31	11	-	6	17	6	3	28
***Caste-system***								
Jatt	35	19	9	7	7	6	7	11
Arain	40	10	12	10	6	3	5	14
Rajput	34	13	6	6	6	4	4	27
Malik	55	8	5	3	8	-	5	18
Sheikh	38	5	5	13	8	-	3	28
Others	43	12	11	6	5	6	3	14
***Economic status***								
Low	44	12	10	5	4	4	5	16
Mid	31	16	8	11	9	6	4	15
High	25	11	11	21	7	7	-	18

* differences in the distributions were statistically significant in all variables; data presented in percentages.

## DISCUSSION

This study provides a comprehensive description of CA prevalent in the Faisalabad population. In the present cohort, the four most common categories of CA were neurological disorders, sensorineural/ear defects, neuromuscular disorders and limb defects, accounting for 69% of the anomalies, which demonstrate the substantial impact of these anomalies resulting in a high disability burden. The previous studies conducted in Sialkot and Rawalpindi districts of Pakistan have also pinpointed neurological anomalies to be the most prevalent CA.^3^,^9^

This study provides a detailed breakdown of CA, categorizing them into major and minor groups, along with their frequencies, proportions and 95% confidence intervals (CI). Some of the extremely rare disorders observed in this population included Bardet-Biedel syndrome, Fragile-X syndrome and MASA syndrome (among neurological disorders), brachydactyly-symphalangism syndrome, macrodactyly, oligodactyly, thumb hypoplasia, radial hemimelia (among limb defects), Fanconi anemia and von Willebrand disease (among blood disorders), tetralogy of Fallot and pulmonary vein stenosis (among congenital heart defects), bladder exstrophy and Bartter syndrome (in urogenital defects), galactosemia and mucopolysaccharidosis (in metabolic defects) and cutis aplasia congenita, ichthyosis and Sjogren’s syndrome (among Others category). This cohort of rare disorders may be further utilized for prospective molecular studies for the identification of new genes, improving diagnostic techniques through next-generation sequencing and to explorer innovative treatment options and targeted therapies.

Given the increased frequency of sporadic cases (66%) compared to familial (34%), the role of environmental and non-genetic factors, such as maternal health, nutritional deficiencies and exposure to teratogens, cannot be ruled out in this population. Sporadic occurrence was common for congenital heart defects (91%) and urogenital defects (86%). Studies conducted in other LMICs have demonstrated the influence of environmental factors on the prevalence of certain CA.^10^,^11^ Faisalabad, known as the industrial hub of Pakistan, particularly for its textile sector, has witnessed increasing environmental pollution due to unregulated discharge of chemical waste and overuse of agrochemicals, leading to a significant accumulation of toxic residues in soil and crops.^12^ Health studies from the region have documented a range of acute and chronic effects associated with chemical exposure, including dermatological conditions, respiratory diseases, gastrointestinal disorders, reproductive health problems and neurological symptoms.^13^,^14^ Newborns and pregnant women are especially vulnerable to the toxic effects of pesticide and chemical exposure.^15^ This may explain high incidence of sporadic cases in few of the main categories of CA. This further warrants the need for maternal healthcare improvement and public health interventions to mitigate controllable risk factors.

Consanguinity has been shown to be a risk factor for the occurrence of CA. With 62% of affected individuals were born to consanguineous parents, especially in blood disorders (83%), the study further accentuates the impact of consanguinity. This is consistent with other research that found a considerable correlation between consanguinity and autosomal recessive illnesses in Pakistan and other regions with high prevalence of consanguinity.^3^,^7^

This study revealed that Toba Tek Singh tehsil had the highest burden of neurological disorders, sensorineural/ear defects and neuromuscular disorders compared to other regions like Faisalabad city (Table-IV). This could be explained by the lower socioeconomic status, malnutrition, and inadequate maternal nutrition which increase susceptibility to congenital and developmental anomalies. The region’s agricultural and semi-industrial profile involves extensive use of pesticides, fertilizers, and possibly contaminated groundwater which are linked to neurodevelopmental and auditory toxicity.^16^ Further, there is restricted access to prenatal screening, perinatal care, and neonatal services can contribute to birth-related complications, hypoxia, or infections that lead to neurological and motor impairments. Further, the rural areas like Toba Tek Singh often have strong cultural traditions of cousin marriages, increasing the likelihood of autosomal recessive disorders many of which present as neurological or neuromuscular conditions.

This study has following strengths. First, it presents a large first-hand data on CA and gives a comprehensive overview of the anomalies prevalent in Faisalabad population, which is useful in elucidating the burden of diseases. The use of standardized classification systems (OMIM, ICD-10) in this study allowed for precise categorization of anomalies. By integrating clinical, demographic, and familial information, the study offers valuable insight into the distribution and determinants of CA across sex, region, and ethnicity. Further, the identification of several rare disorders adds to the genetic documentation of underreported conditions in the local population. The detailed distribution of CA along various variables is important in understanding the inequalities across the ethno-demographic factors.

### Strengths of the study:

Despite these strengths, several areas warrant further investigation. Molecular genetic studies, including next-generation sequencing, are needed to confirm hereditary cases and identify novel pathogenic variants/gene(s), particularly in families with rare disorders. Detailed assessments of maternal health, nutritional status, and exposure to environmental teratogens would clarify the role of non-genetic factors, especially in sporadic cases. Longitudinal follow-up of affected individuals could help evaluate survival and disability outcomes.

### Limitations:

The cross-sectional design of this study limits the ability to establish causal relationships between risk factors and CA. This study does not report population-based prevalence of CA. There were no data included from aborted pregnancies or stillbirths, possibly underestimating CA prevalence.

These findings have important public health implications. The high prevalence of neurological disorders underscores the need for early diagnosis and intervention programs. Families with a history of rare conditions may benefit from genetic counseling to understand risks and preventive measures including controllable risk factors of maternal exposure. The potential association of high consanguinity rate with occurrence of CA should be highlighted. This study also emphasizes the necessity of better prenatal care, population-based screening initiatives and public health education in order to lessen the prevalence of CA in Pakistan.

## CONCLUSIONS

This study highlights the significant burden of CA in the Faisalabad division, with neurological disorders being the most prevalent. The high rate of consanguinity and predominance of sporadic cases suggest that both genetic and environmental factors may contribute to the etiologies of CA in this population.

### Authors’ Contribution:

**SM:** Conceived, designed and supervised the study

**SS, ZB, AR and SN:** Data collection, curation, clinical evaluation, and statistical analysis

**SM:** Responsible and accountable for the accuracy and integrity of data.

All authors have read and approved final version of the manuscript.
